# Development and Psychometric Examination of a Short-Form Dementia Attitudes Scale

**DOI:** 10.1093/geroni/igaa057.908

**Published:** 2020-12-16

**Authors:** Alexandria Ebert, Jenessa Steele, Julie Hicks Patrick

**Affiliations:** 1 West Virginia University, Morgantown, West Virginia, United States; 2 Radford University, Radford, Virginia, United States

## Abstract

With the increase in research related to people directly and indirectly affected by Alzheimer’s Dementia and related disorders (ADRD), there is a need for short, psychometrically-sound instruments to assess the variety of influences on the quality of life for the person with dementia (pwd) and their care partners. We sought to develop and assess a short version of the Dementia Attitudes Scale (DAS; O’Connor & McFadden, 2010) for use in such endeavors. Using a sample of 321 adults (Mage = 39.7, range 20 to 70 yrs; 47% female), we surveyed a host of individual characteristics (e.g., age, personality, experience with pwd) and several current measures of attitudes about pwd. Ten days later, 162 of these adults completed a retest of the DAS. Results of a confirmatory factor analysis supported the 2-factor solution reported by O’Connor and McFadden. Internal consistency was acceptable for the Comfort factor (Time 1alpha = .83; T2 alpha = .82) and for the Knowledge factor (Time 1 alpha = .86; Time 2 alpha = .87). These estimates are similar to those in the original report. Due to limitations of coefficient alpha we also used structural equations to examine the regression weights for each item as a function of chronological age and experience. These analyses showed that the factor structure was robust when considering these individual characteristics. Finally, we compared different scale lengths using a series of ROC curves. We discuss our results in the context of providing brief and psychometrically-sound measures for use in large survey studies.

